# Microbial ecology of selected traditional Ethiopian fermented products

**DOI:** 10.3389/fmicb.2025.1570914

**Published:** 2025-06-02

**Authors:** Carmen Sanz-López, Michela Amato, Daniel Torrent, Marta Borrego, Mathewos Anza, Mesfin Bibiso, Nubia Grijalva-Vallejos, Cristina Vilanova, Manuel Porcar, Javier Pascual

**Affiliations:** ^1^Darwin Bioprospecting Excellence S.L., Paterna, Spain; ^2^Department of Chemistry, College of Natural and Computational Sciences, Wolaita Sodo University, Wolaita Sodo, Ethiopia; ^3^Quiitos S.A.S, San Antonio de Ibarra, Ecuador; ^4^Institute for Integrative Systems Biology I2SysBio, University of Valencia, CSIC, Paterna, Spain

**Keywords:** Ethiopian fermented products, traditional fermented foods and beverages, microbial ecology, metataxonomics, culturomics

## Abstract

The consumption of traditional fermented foods and beverages plays an important role in the diet of Ethiopia, providing significant nutritional and health benefits to the local population. The present study aimed to investigate the microbial ecology and diversity of nine types of fermented products. These include two foods (*Kotcho* and *Injera*), one food condiment (*Datta*), and six beverages (*Tej*, *Tella*, *Cheka*, *Kinito*, *Borde*, and *Shamita*). A combination of metataxonomic and culturomic approaches was used to achieve a comprehensive characterization of the bacterial communities, together with a thorough physicochemical characterization of the fermented products. This study provides one of the most comprehensive microbial characterizations of a wide selection of Ethiopian fermented products, highlighting that some bacterial species involved in the fermentation processes could contribute to the safety and nutritional quality of fermented foods and, based on previous studies, could also play a key role in enhancing their potential probiotic properties.

## 1 Introduction

In many geographical regions of the world, traditional fermented foods and beverages play a vital role in the diet. This is particularly evident in developing countries, where these fermented products are often used as a safe and easily storable source of nutrients, vitamins and probiotics. Traditional fermented products are usually made by hand using traditional methods and local ingredients. The main purposes of fermentation are to improve the sensory properties of foods and beverages, extend their shelf life, displace undesirable microorganisms, increase their nutritional value and detoxify raw materials (Marco et al., 2017). Most traditional fermentation practices involve spontaneous processes in which raw materials are fermented at ambient temperatures, often uncovered, allowing exposure to environmental microorganisms. Additionally, microbial starters from a previously fermented batch are often incorporated, which helps to initiate fermentation and enhance microbial diversity (Wedajo Lemi, 2020). Lactic acid bacteria (LAB), together with yeasts, are usually the predominant microorganisms involved in the fermentation process of these products (Holzapfel et al., 1995). Certain LAB species are characterized by their ability to exert probiotic effects, thereby improving the health and well-being of consumers (Akalu et al., 2017). In addition to LABs, other bacterial groups such as *Bacillus* and related genera as well as acetic acid bacteria may also contribute to the fermentation process and may have probiotic properties, improving gut health, immune modulation and nutrient bioavailability (Guarner and Schaafsma, 1998). Therefore, the importance of traditional fermented foods and beverages to human nutrition and health is clear, especially in regions where foodborne diseases are prevalent and access to refrigeration is limited. The Ethiopian diet is characterized by a unique diversity of traditional fermented foods and beverages, which play an important role in the dietary habits of the native population (Mulaw and Tesfaye, 2017; Hotessa and Robe, 2020; Wedajo Lemi, 2020). Factors such as the type and origin of the raw material influence the initial microbial community involved in fermentation. In addition, physico-chemical parameters of the fermentation process, such as temperature, pH and moisture content, play a crucial role in shaping microbial diversity and activity. To date, microbiological studies on traditional Ethiopian fermented products such as *Kinito* (Abawari, 2013), *Shamita* (Akalu et al., 2017), *Borde* (Ashenafi and Mehari, 1995), *Kotcho* (Akalu et al., 2017), *Tella* (Berhanu, 2014), and *Datta* (Idris and Ashenafi, 2001) have mainly relied on cultured bacteria using classical approaches. However, given the challenges associated with culturing certain bacterial species using conventional culture media, advanced metataxonomic approaches are essential to gain access to the full microbial diversity and improve our understanding of their presence in traditional fermented products. These methods allow a more detailed characterization of microbial diversity and functional potential, overcoming the limitations of culture-dependent techniques. The integration of metataxonomic and culture-dependent techniques is necessary to gain precise insights into the role of microorganisms in the physicochemical and organoleptic properties of fermented foods, as well as their benefits as sources of potential probiotic microorganisms.

This study investigated the microbial ecology of nine Ethiopian fermented products, including two foods (*Kotcho* and *Injera*), one food condiment (*Datta*), and six beverages (*Tej*, *Tella*, *Cheka*, *Kinito*, *Borde*, and *Shamita*). To our knowledge, this is one of the most extensive microbial characterizations of Ethiopian fermented products to date. Biochemical, culturomic and state-of-the-art metataxonomic methods were used to achieve a holistic understanding of the microorganisms involved in fermentation processes, including the assessment of the safety of fermented foods and beverages from a sanitary point of view, in addition to their potential probiotic benefits and implications for human health and nutrition.

## 2 Materials and methods

### 2.1 Sample collection

**Figure 1:**
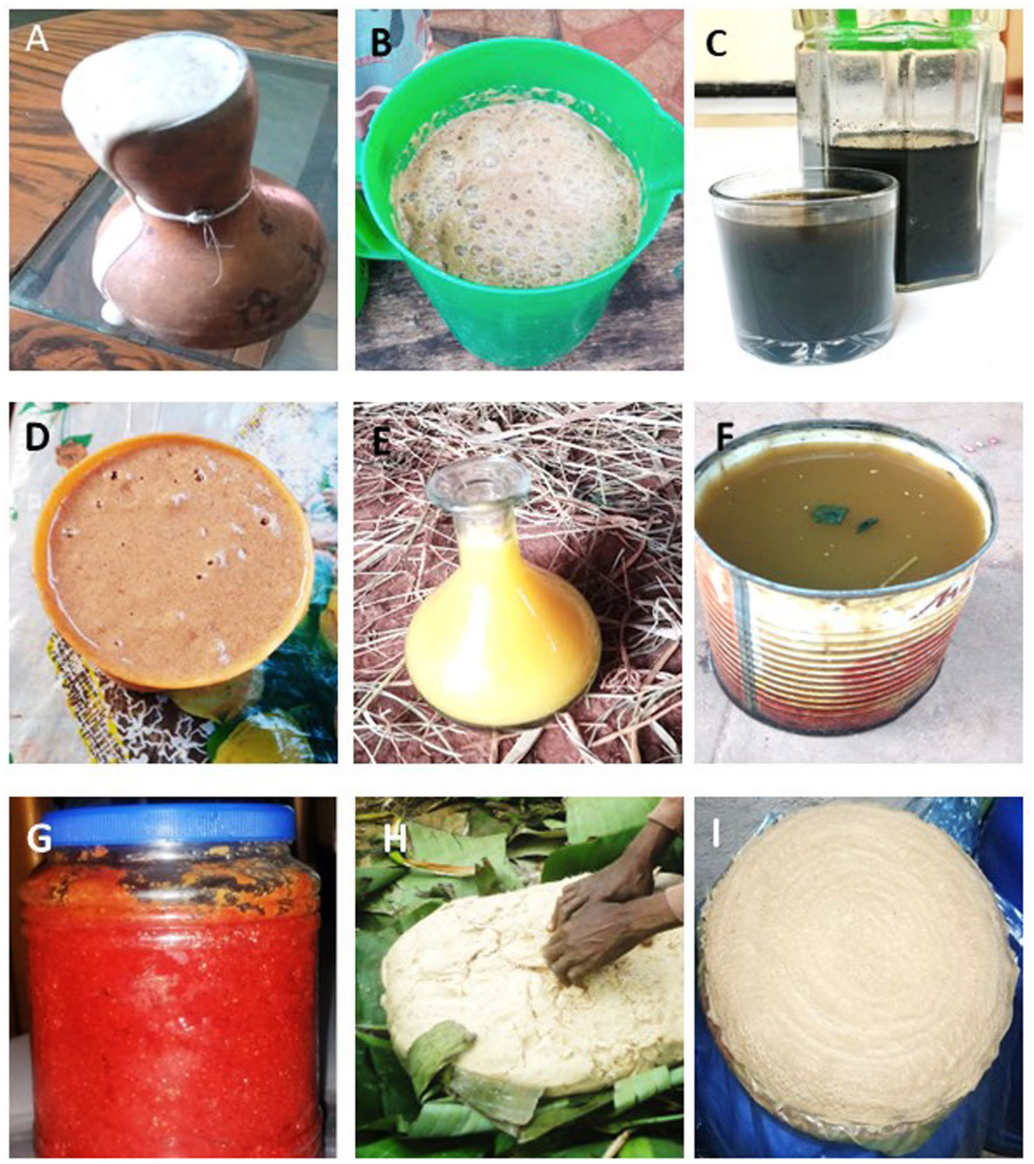
Traditional fermented beverages **(A)** *Borde*, **(B)** *Cheka*, **(C)** *Kinito*, **(D)** *Shamita*, **(E)** *Tej*, **(F)** *Tella*; food condiment **(G)** *Datta*; and foods **(H)** *Kotcho* and **(I)** *Injera* analyzed in this study.

A sampling campaign of traditional Ethiopian fermented foods and beverages was conducted in the Wolaita zone of southern Ethiopia in May 2022. This region was selected due to its rich tradition of fermentation practices and the cultural importance of these products in Ethiopian households. Although the campaign focused solely on the Wolaita zone due to its proximity to Wolaita Sodo University, a comparative analysis of the fermentation processes and ingredients used in Wolaita, Konso and Dirashe was carried out. This analysis showed that the fermentation methods and ingredients were consistent across these areas, making the results representative and applicable beyond the study area. The campaign was approved by the Ethiopian Biodiversity Institution, ensuring compliance with ethical and regulatory standards. A total of nine fermented products were collected during the campaign: *Kotcho*, *Datta*, *Injera*, *Tej*, *Tella*, *Cheka*, *Kinito*, *Borde*, and *Shamita* (Figure 1). These products were chosen to represent a diverse range of food types and beverages widely consumed in the region, providing a comprehensive basis for studying microbial ecology and fermentation dynamics. Upon collection, the samples were immediately shipped from the Organic Chemistry Laboratory, Department of Chemistry, Wolaita Sodo University, Ethiopia, to Darwin Bioprospecting Excellence, Valencia, Spain, kept at 4°C to prevent microbial alteration. Samples were processed in parallel for metataxonomic analysis, culturomics and physicochemical profiling. In this study, a single sample of each of the nine Ethiopian fermented products was analyzed. This approach was chosen to provide a broad overview of the microbial ecology of different types of traditional fermented foods and beverages, rather than examining microbial variability within multiple batches of the same product.

### 2.2 Biochemical analysis of the samples

The concentrations of free sugars (glucose, fructose, and sucrose), ethanol, lactic acid, acetic acid, citric acid, gluconic acid, and glutamic acid were quantified to determine the biochemical profile of the samples. The biochemical compounds analyzed in this study-such as organic acids and ethanol-were selected because they are the main compounds normally involved in microbial fermentation processes. The pH of the samples was also measured because its direct relation with the fermentation process. These parameters are key indicators of fermentation quality, as they reflect microbial activity, metabolic pathways, and the overall stability of the fermented products. These compounds were measured using a spectrophotometric colorimetric method with the Y15 automated instrument (BioSystems, Barcelona, Spain). The reagent kits used included sucrose/D-glucose/D-fructose (ref. 12,819), ethanol (ref. 12,847), L-lactic acid (ref. 12,802), acetic acid liquid (ref. 12,930), citric acid (ref. 12,825), gluconic acid/gluconolactone (ref. 12,811), and L-glutamic acid (ref. 12,830). Samples likely to contain proteins (*Borde*, *Shamita*, *Cheka*, *Datta*, and *Injera*) were pre-treated with the Carrez kit (ref. 12,837) to avoid interferences in the measurements. The solid samples were diluted with water, and appropriate calculations were then performed to obtain the concentration of each molecule.

### 2.3 Extraction of microbial metagenomic DNA and quantification

DNA was extracted using the DNAeasy PowerSoil kit (QIAGEN, Hilden, Germany). The use of this commercial DNA extraction kit reduced experimental variability and ensured optimal DNA concentration and integrity. Once extracted, the DNA was quantified using Qubit dsDNA High Sensitivity technology (Invitrogen, California, USA), a fluorometric method that allows accurate quantification of DNA in environmental samples at low concentrations. Additionally, extracted DNA quality was analyzed by agarose gel electrophoresis (1.2% w/v).

### 2.4 Illumina sequencing of the 16S rRNA gene amplicons and bioinformatic analysis

After confirming the presence of DNA in the samples, libraries were prepared following Illumina’s standard protocol. Amplicons were sequenced using the Illumina MiSeq platform (2×300 bp). A detailed description of the amplification protocol and library preparation is available in Satari et al. (2020). Briefly, Metagenomic DNA was isolated using the DNeasy PowerSoil Pro Kit (QIAGEN, Hilden, Germany), and its concentration was determined with the QUBIT dsDNA HS-High Sensitivity Kit (Invitrogen, CA, USA). The V3-V4 hypervariable regions of the 16S rRNA gene were then amplified via PCR following the Illumina MiSeq protocol, utilizing the recommended forward (5′-TCGTCGGCAGCGTCAGATGTGTATAAGAGACAGCCTACGGGNGGCWGCAG-3′) and reverse primers (5′-GTCTCGTGGGCTCGGAGATGTGTATAAGAGACAGGACTACHVGGGTATCTAATCC-3′) (Klindworth et al., 2013). The amplification was conducted using the KAPA HiFi HotStart ReadyMix PCR kit (KK2602) under the following conditions: initial denaturation at 95°C for 3 min, 25 cycles of denaturation (95°C for 30 s), annealing (55°C for 30 s), and extension (72°C for 30 s), followed by a final extension step at 72°C for 5 min. Subsequently, Illumina sequencing adapters and dual-index barcodes (Nextera XT index kit v2, FC-131-2001) were incorporated into the amplicons. Prior to sequencing, libraries were normalized and pooled. To enhance sequencing accuracy, the indexed amplicon pool was loaded onto the MiSeq reagent cartridge v3 (MS-102-3003) with a 10% PhiX control spike-in. Paired-end sequencing (2 × 300 bp) was performed using the Illumina MiSeq platform. For each fermented product, a single technical replicate was analyzed. Appropriate negative (blank extraction) and positive (previously characterized sample) controls were included in the DNA extraction, library preparation and bioinformatics workflow to ensure data reliability and to account for potential contamination.

Raw Illumina sequences were processed using Qiime2 (v. 2021.2.0) (Bolyen et al., 2019). Sequence quality was assessed via the Demux plugin, and the DADA2 pipeline within Qiime2 was employed for trimming, merging, chimera filtering, and identifying amplicon sequence variants (ASVs) with >99.9% similarity. Taxonomic classification of each ASV was performed using the classify-Sklearn module with the Greengenes2 reference tree (v. 2022.10) (McDonald et al., 2024) as the reference database. This version of the taxonomic database uses the old names of some recently updated taxa (https://lpsn.dsmz.de/). Data analysis and visualization were conducted using the phyloseq R package (v. 1.48.0) (McMurdie and Holmes, 2013) and ggplot2 (v. 4.4.0) (Wickham et al., 2023). Alpha diversity analyses were performed on ASV counts rarefied to the smallest sample size using the rarefy_even_depth function in phyloseq. PCoA plots were generated with the plot_ordination function, also from phyloseq, using the Bray–Curtis dissimilarity metric as the distance measure. The UPGMA dendrogram was constructed using the ggdendro (v. 0.2.0) (De Vries and Brain, 2020) and ape (v. 5.8) (Paradis et al., 2024) R packages, based on a Bray–Curtis dissimilarity matrix and hierarchical clustering using the “average” linkage method. Heat maps were generated using the amp_heatmap function from the ampvis2 R package (v. 2.7.2) (Andersen et al., 2018) and the pheatmap R package (v. 1.0.12) (Kolde, 2019).

### 2.5 Isolation of bacterial strains

Upon arrival at the laboratory, samples were homogenized by combining 1 g of each sample with 1 mL of sterile saline (NaCl, 0.9%) and serially diluted to 10^−7^. Then, 50 μL of the 10^−2^ to 10^−7^ dilutions were plated on seven Petri dishes containing different culture media, including R2A (DSMZ medium 830), TSB (DSMZ medium 545), *Gluconobacter oxydans* medium (DSMZ medium 105), MRS (DSMZ medium 11), lithium propionate MRS (DSMZ medium 11 supplemented with 0.2% lithium chloride and 0.5% v/v propionic acid), and *Bifidobacterium* medium (DSMZ medium 58). These culture media were formulated to support the growth of a wide range of bacterial species involved in the fermentation of many types of foods and beverages, ensuring comprehensive coverage of the microbial diversity present in samples. The Petri dishes were then incubated for five days at 30°C under aerobic (R2A, TSB, *Gluconobacter oxydans* medium) or microaerophilic conditions using the candle jar method (MRS, lithium propionate MRS, *Bifidobacterium* medium). Individual colonies were selected based on color, shape, size, height, rim, surface area, and opacity, then grown on fresh media to obtain pure cultures. Isolates were cryopreserved in 20% glycerol and stored at −80°C for future use.

### 2.6 Molecular identification of isolates

Genomic DNA was extracted from the isolates using the DNA Microbiome Extraction Protocol of the Mag-Bind Bacterial DNA 96 Kit (Omega Bio-tek, Norcross, Georgia, USA) and the Auto-Pure 96 equipment (ALLSHENG, Hangzhou, China). PCR was performed with the commercial mix NZYTaq II 2x Green Master (NZYTech, Lisbon, Portugal) using the Mastercycler® nexus thermocycler (Eppendorf, Hamburg, Germany) according to the manufacturer’s recommendations. The universal primers 8F (5′-AGAGTTTGATCCTGGCTCAG-3′) (Edwards et al., 1989) and 1492R (5′-GGTTACCTTGTTACGACTT-3′) (Stackebrandt and Liesack, 1993) were used to amplify the 16S rRNA gene. The following PCR conditions were used: initial denaturation (95°C for 5 min), amplification (24 cycles of denaturation at 94°C for 15 s, annealing at 48°C for 15 s, and extension at 72°C for 90 s), and a final extension (72°C for 5 min). After PCR, the amplicons were precipitated overnight at −20°C in a mixture of isopropanol (1:1, vol/vol) and potassium acetate (1:10, vol/vol) (3 M, pH 5). The DNA was then centrifuged at 12,000 rpm for 10 min, washed with 70% ethanol, and resuspended in 10 μL of sterile Milli-Q water. Amplicons were labeled with the BigDye® Terminator v3.1 Cycle Sequencing Kit (Applied Biosystems, Carlsbad, CA, United States) and sent to the SCSIE (Serveis Centrals de Suport a la Investigació Experimental) of the University of Valencia (Spain) for Sanger sequencing of the partial 16S rRNA gene using the universal primer 8F. The PCR products were verified for integrity and quality using agarose gel electrophoresis (1.2% w/v) before sequencing.

All resulting sequences were edited to remove low-quality base calls. Taxonomic identification was performed using the BLASTn tool and the 16S ribosomal RNA sequences (Bacteria and Archaea) database (NCBI). Finally, clones of each sample were dereplicated using the MEGA-X software (https://www.megasoftware.net/) to compare each partial 16S rRNA sequence to the rest of the strains with the same identification. This step was performed to avoid overestimating the culturable diversity, as bacterial clones of the same species are not relevant for the microbial collection.

## 3 Results

### 3.1 Physicochemical characterization of fermented foods and beverages

**Figure 2:**
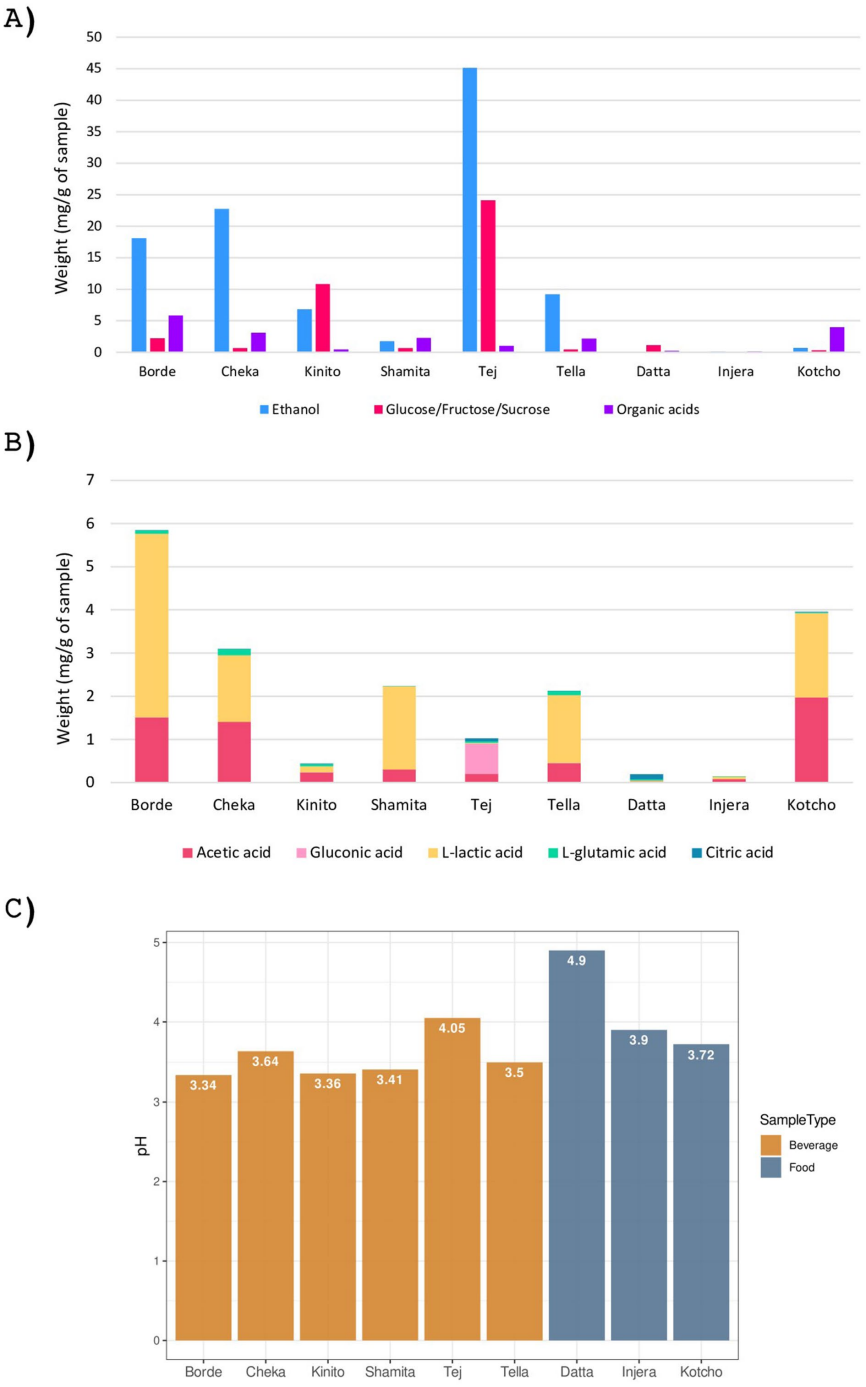
Chemical characterization of the nine fermented products analyzed. **(A)** Biochemical composition of the analyzed samples, expressed in milligrams of compound per gram of sample. The molecules represented include sugars, ethanol, and organic acids. **(B)** Composition of organic acids in the analyzed samples, expressed in milligrams of compound per gram of sample. The organic acids analyzed are acetic, citric, lactic, gluconic, and glutamic acids. **(C)** pH values of the analyzed samples.

In this study, a comprehensive physicochemical characterization was conducted on two fermented foods, one condiment and six fermented beverages indigenous to Ethiopia (Figure 2). Acidic pH values below 5.0 were observed in all the fermented foods and beverages. Among the beverages, *Borde* and *Kinito* exhibited the most acidic pH values, with readings of 3.34 and 3.36, respectively. The most acidic fermented food was *Kotcho*, with a pH of 3.72.

The analysis revealed that all the fermented beverages contained alcohol, with *Tej* exhibiting the highest ethanol content at 45.1 mg/g, followed by *Cheka* (22.7 mg/g) and *Borde* (18.0 mg/g). The lowest ethanol content was observed in *Shamita*, with a value of 1.7 mg/g. In the case of fermented foods, all three had ethanol levels below 0.7 mg/g (Figure 2C).

The free sugar content of the samples was characterized after the fermentation process by analysing the combined levels of glucose, fructose, and sucrose (Figure 2A). These sugars play a crucial role in the production of fermented foods and beverages, serving as primary substrates for fermentation by microorganisms such as yeast and bacteria. Among the beverages, the highest sugar content after fermentation was found in *Tej* (24.1 mg/g), followed by *Kinito* (10.8 mg/g). Conversely, *Cheka*, *Shamita*, and *Tella* had low sugar content (<0.7 mg/g). The fermented foods also exhibited low sugar content, with *Datta* having the highest (1.1 mg/g) and *Injera* the lowest (0.03 mg/g).

Organic acids are synthesized by microorganisms during fermentation as by-products of their catabolism. The presence of organic acids in traditional Ethiopian fermented foods and beverages plays a crucial role in enhancing their nutritional value and safety. Among the beverages, *Borde* had the highest amount of organic acids (5.8 mg/g), followed by *Cheka* (3.1 mg/g) (Figure 2B). In contrast, *Kinito* exhibited a low content of organic acids (Figure 2B). Lactic acid was the predominant organic acid in all fermented beverages except *Kinito*, where acetic acid was significant, and *Tej*, where gluconic acid followed by acetic acid were the main organic acids. Notably, *Tej* also had the highest amount of citric acid, making it the drink with the most diverse organic acid profile. All beverages except *Shamita* contained glutamic acid. Among the foods and condiments, *Kotcho* had the highest content of organic acids, with significant amounts of lactic and acetic acids. In contrast, both *Injera* and *Datta* had only residual amounts of organic acids; however, despite its low overall concentration, *Datta* showed a diverse profile, including citric, glutamic, lactic, and acetic acids.

When all the nutritional data were analyzed together, the two fermented foods and the condiment were not closely related (Supplementary Figure S1). *Kotcho* was more similar to *Shamita*, sharing a comparable profile of ethanol, free sugars, and organic acids (Figure 2). Among the fermented beverages, *Borde* and *Cheka* were the most similar, while *Tej* and *Shamita* were the most different. Overall, *Datta* and *Injera* were found to have the most distinct nutritional profiles, primarily due to their reduced or absent levels of ethanol, free sugars, and organic acids (Supplementary Fig. S1).

### 3.2 Metataxonomic characterization of the fermented foods and beverages

In this study, the bacterial communities present in various fermented products were analyzed using next-generation sequencing of metagenomic 16S rRNA sequences. The aim was to elucidate the complete bacterial community in each product, the role of microorganisms in fermentative processes, and their biological functions from a nutritional perspective. A significant number of sequences were obtained from all samples, ranging from 60,070 high-quality reads in *Cheka* to 22,362 in *Tella* for fermented beverages, and from 50,756 in *Kotcho* to 42,113 in *Injera* for fermented foods (Supplementary Figure S2A). It was observed that the rarefaction curves for all fermented products analyzed reached a plateau, indicating that the full diversity of taxa present in the samples was captured (Supplementary Figure S2B).

**Figure 3:**
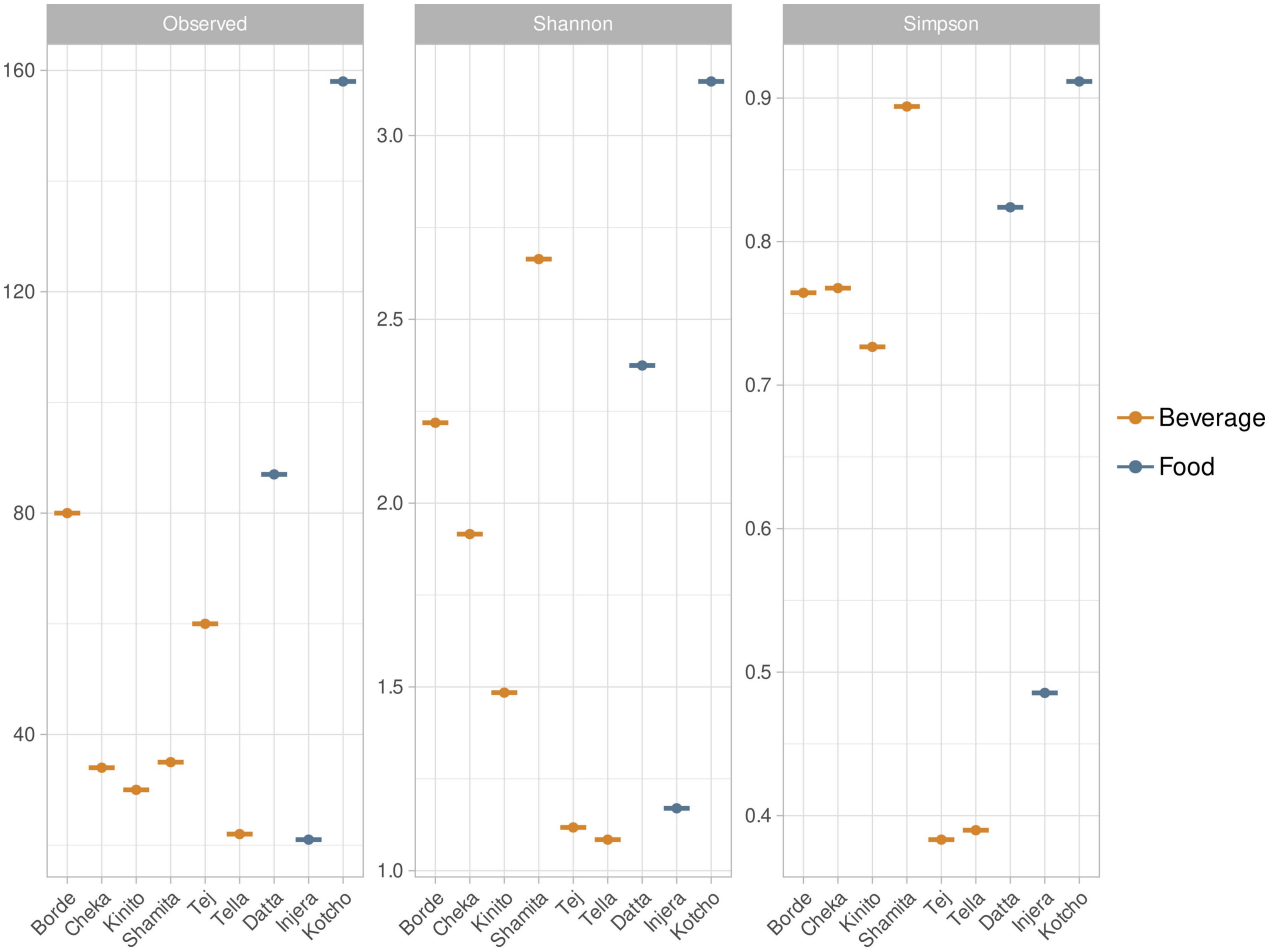
Alpha diversity values for traditional fermented beverages and foods of Ethiopia. This figure presents the alpha diversity values for each of the traditional fermented beverages and foods of Ethiopia. The calculated values include species richness (S) which quantifies the total number of different bacterial species (Amplicon Sequence Variant, ASV) present in the fermented products. Shannon Index (H′) reflects the diversity and evenness of species distribution and Simpson Index (D) which expresses the probability that two individuals belong to the same species.

The fermented drink with the highest number of bacterial species was *Borde*, with 80 ASVs, followed by *Tej* (Figure 3). Conversely, *Shamita* and *Borde* exhibited the highest Shannon and Simpson diversity index values among the fermented beverages, indicating that *Shamita* had a high number of species with balanced relative abundances. The fermented drinks with the lowest bacterial species richness were *Shamita* (35 ASVs), *Cheka* (34 ASVs), and *Kinito*. Among the fermented foods, *Kotcho* had the highest bacterial diversity in terms of richness and distribution of taxa abundances (158 ASVs). In contrast, *Injera* had the lowest taxa richness (21 ASVs). Richness refers to the total number of different taxa present in the community, while diversity indices, such as the Shannon or Simpson indices, provide a broader measure of biodiversity by taking into account how evenly individuals are distributed among taxa.

**Figure 4:**
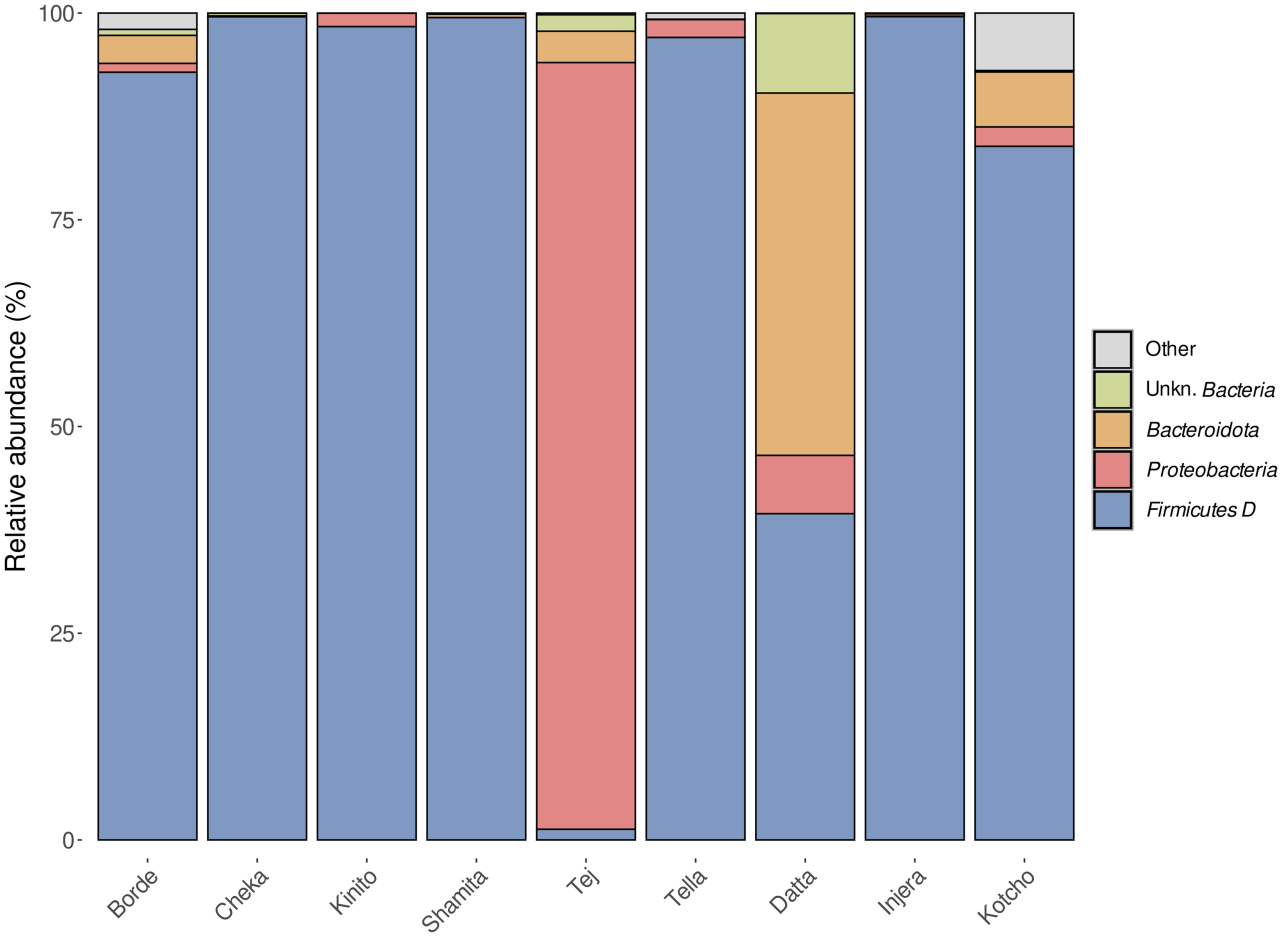
Relative abundance of bacterial phyla identified in the nine fermented products analyzed.

The bacterial communities found in all the fermented beverages analyzed were predominantly composed of members of the phylum *Firmicutes* (now known as *Bacillota*), except for *Tej*, which was characterized by the dominance of members of the phylum *Proteobacteria* (now known as *Pseudomonadota*), followed by *Bacteroidota* (Figure 4). In the case of fermented foods, the bacterial community present in *Injera* was almost completely dominated by *Firmicutes*. In contrast, *Kotcho* contained members of the phyla *Bacteroidota* and *Proteobacteria* in addition to *Firmicutes*. *Datta* had the most distinct microbial community, with *Bacteroidota* as a dominant phylum, followed by *Proteobacteria* and *Firmicutes*.

**Figure 5:**
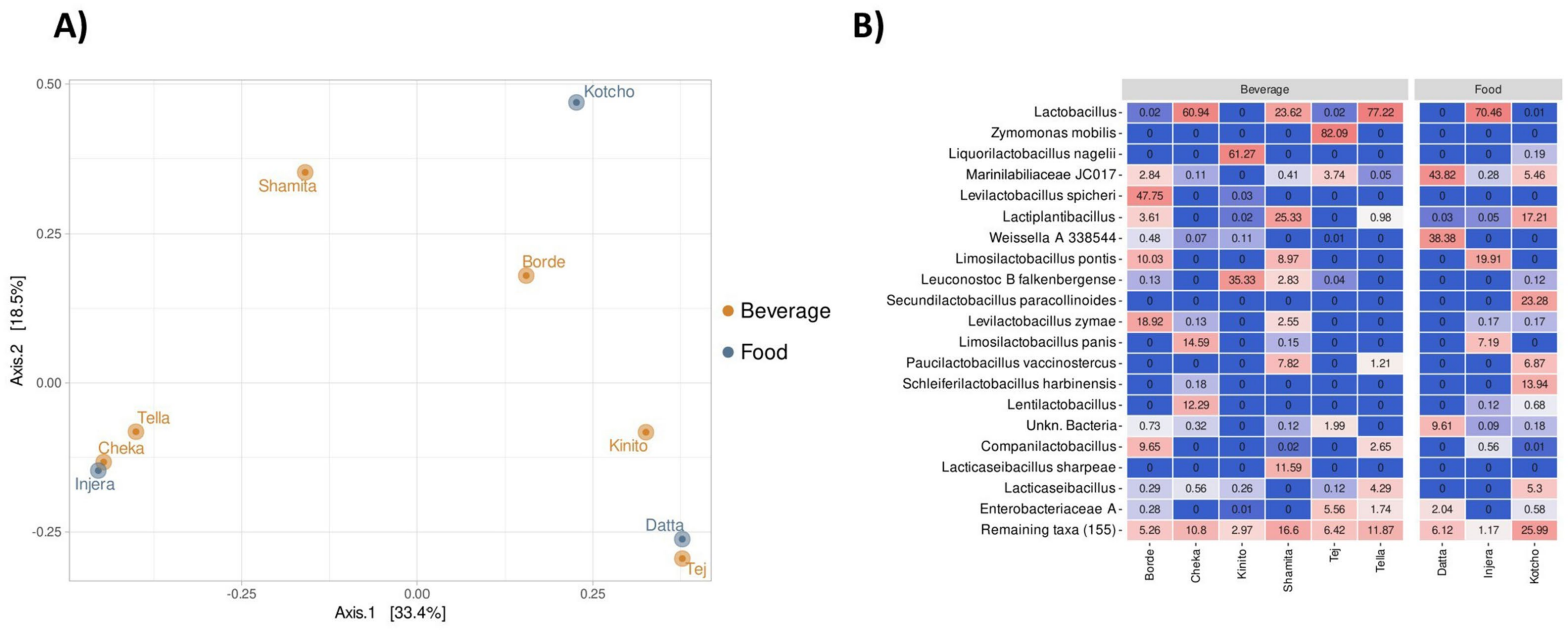
Analysis of nine fermented products from Ethiopia. **(A)** Principal Coordinates Analysis (PCoA) at the species level for the nine fermented products. **(B)** Heatmap showing the relative abundance (%) of the 20 most prevalent species across the nine fermented products.

The bacterial communities in fermented beverages, condiment and foods showed differences at the genus level (Figure 5A; Supplementary Table S1). The fermented food *Injera* had a similar taxonomic composition to the beverages *Tella* and *Cheka*. Specifically, these three products were dominated (>60% of the total community) by members of the genus *Lactobacillus* (Figure 5B). Representatives of other genera within the *Lactobacillaceae* family were also present in these products, but in lower relative abundance. In the case of *Borde*, the dominant genus was *Levilactobacillus* (68.3%), mainly represented by the species *Levilactobacillus spicheri* and *Levilactobacillus zymae*, as well as *Limosilactobacillus ponticus* (10.0%). In *Kinito*, the dominant taxa were primarily *Liquorilactobacillus nagelii* (61.1%) and *Leuconostoc falkenbergense* (35.3%). In *Kotcho*, the dominant taxa were *Secundilactobacillus paracollinoides* (23.3%), *Lactiplantibacillus* spp. (17.2%), and *Schleiferilactobacillus harbinensis* (13.9%). *Shamita* was characterized by high levels of *Lactiplantibacillus* spp. (25.3%), *Lactobacillus* spp. (23.6%), and *Lacticaseibacillus sharpeae* (21.3%). The bacterial community of the alcoholic beverage *Tej* was dominated by members of *Zymomonas mobilis*, which represented 82.1% of the total bacterial community. *Datta* exhibited a more diverse taxonomic profile, enriched with members of lineage JC017 of *Marinilabiliaceae* (43.8%) and representatives of *Weissella* (38.4%).

### 3.3 Bacterial isolation from fermented products

**Figure 6:**
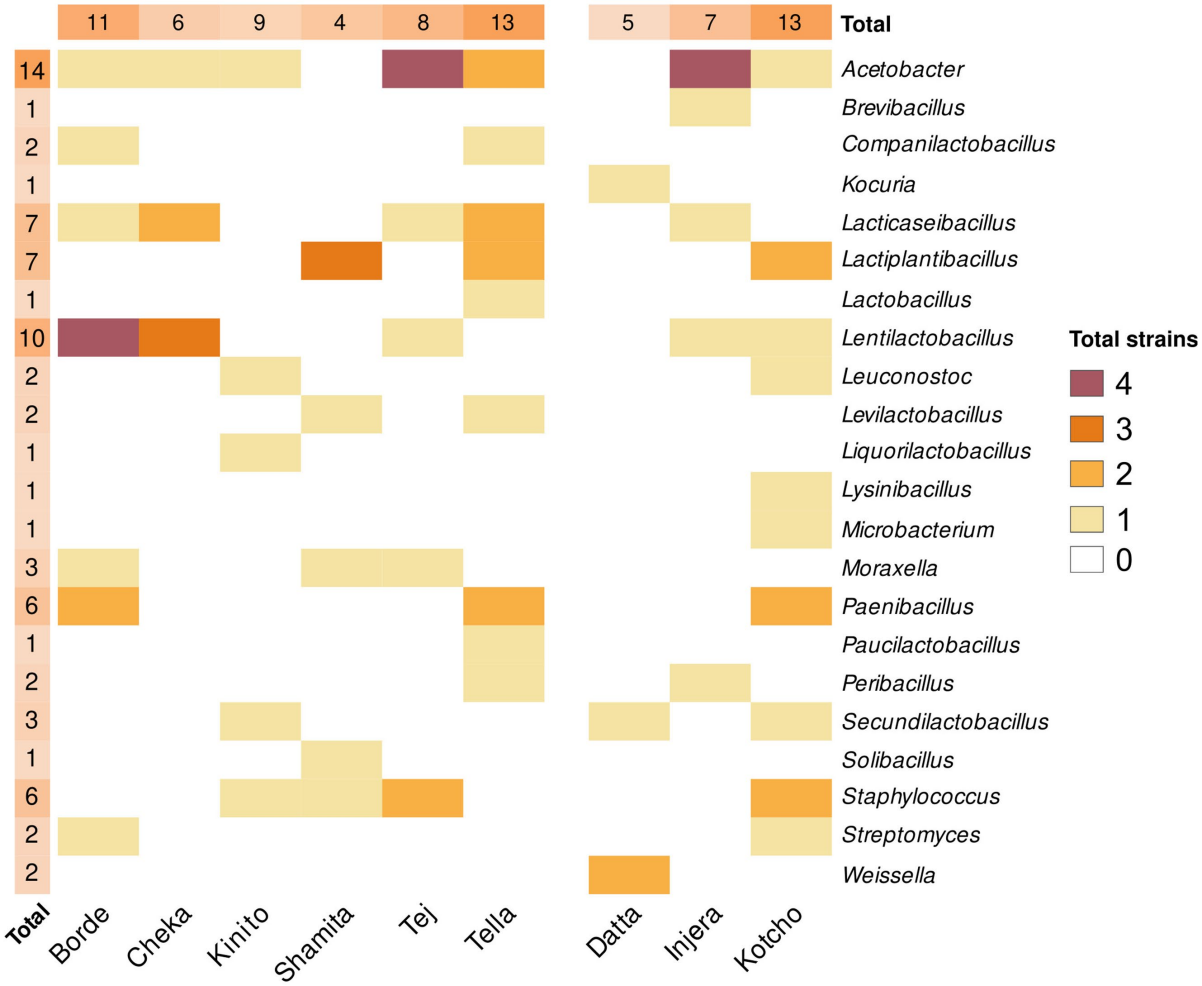
Heatmap of genera isolated from each sample. This heatmap illustrates the number of different genera isolated from each sample. The genus level of strains was determined by partial 16S rRNA gene sequencing of isolates. The color score, ranging from 4 to 0, indicates the number of different species belonging to the same genera isolated in each sample, with 4 representing the highest number of different species and 0 the lowest. The symbol “*” highlights genera isolated by culture techniques but not detected in the metataxonomic analysis of the samples. Only genera with a relative abundance higher than 0.001% were considered detected.

A total of 200 different colonies were isolated from the nine processed fermented products based on their morphology, texture, brightness, and colour. Of these, 79 were identified as different clones based on the partial sequence of the 16S rRNA gene (Figure 6; Supplementary Table S2). Specifically, twenty-six strains were isolated from fermented foods and condiments and 53 strains were isolated from beverages. The isolates belonged mainly to the phylum *Bacillota*, with 34 isolates, followed by *Pseudomonadota* with eight isolates, and one strain from the phylum *Actinomycetota*. The genus Acetobacter was the most abundant and widespread in the samples analyzed. Specifically, 14 different strains were isolated, with at least one representative in all the fermented products analyzed, except *Datta* and *Shamita* (Figure 6). The products with the highest diversity of *Acetobacter* strains were *Tej* and *Injera*. Specifically, *Acetobacter fabarum* was the most common species found in *Kinito*, *Tej*, *Tella*, and *Injera*, while *Acetobacter indonesiensis* was found in *Cheka*, *Tej*, *Tella*, and *Injera*. The next most common genera were *Lentilactobacillus* with 10 strains, followed by *Lacticaseibacillus* and *Lactiplantibacillus* with seven strains each. The beverages with the highest number of isolated strains were *Borde* and *Tella*, both with 13 strains. In *Borde*, the strains were mainly from the genus *Lentiplantobacillus*, while in *Tella* they were mainly from the genera *Acetobacter*, *Lacticaseibacillus*, *Lactiplantibacillus*, and *Paenibacillus*. Conversely, the drink with the lowest diversity of bacterial strains was *Shamita*, with only seven isolates.

Among the fermented foods analyzed, the highest number of isolated bacterial strains was obtained from the *Kotcho* sample (15 different strains), with the most representative genera being *Lactiplantibacillus*, *Paenibacillus*, and *Staphylococcus*. The fermented product with the lowest diversity of isolated strains was *Datta* (four strains), with members of the genera *Weissella*, *Secundilactobacillus*, and *Kocuria*. Unfortunately, bacteria with less than 99.5% sequence similarity to their closest type strains could not be isolated in pure culture. This result shows that the isolated strains do not represent new taxa, and that the isolation focused on already described taxa.

## 4 Discussion

Traditional fermented foods, condiments and beverages constitute an essential part of the Ethiopian diet and culture. Despite studies, mostly culture-dependent, aimed at understanding the microbial diversity involved in traditional fermented products, comprehensive research based on state-of-the-art high-throughput sequencing techniques remains limited (Franz et al., 2014; Tafere, 2015). The objective of the present study was to investigate the taxonomic and functional bacterial diversity in seven fermented beverages, two foods and one condiment. To this end, metataxonomic and culturomic techniques were employed in conjunction with a comprehensive physicochemical characterization of the fermented products. To the best of our knowledge, this study is the first to analyze the widest diversity of Ethiopian traditional fermented products using both culture-dependent and culture-independent techniques. The results for each fermented product analyzed are discussed in the light of current knowledge.

### 4.1 Borde

*Borde* is mainly consumed in the southern and western regions of Ethiopia and is a low-alcohol fermented beverage made from cereals such as maize, barley, wheat, finger millet, sorghum and/or tef, resulting in a thick, sweet–sour drink with a whitish grey to brown colour (Wedajo Lemi, 2020).

The pH of the sample in this study was found to be slightly lower than the pH of the samples analyzed by other authors, namely 3.84 to 4.1 (Ashenafi and Mehari, 1995; Abegaz et al., 2002). The presence of ethanol in the sample may be due to heterofermentative lactic acid bacteria (Wedajo Lemi, 2020).

The most abundant microorganisms identified were LABs, in particular *Levilactobacillus spicheri*, *Levilactobacillus zymae* and *Limosilactobacillus pontis*, which was consistent with the results of previous studies (Abegaz, 2007). These LABs identified in Borde, could contribute to the preservation and sensory characteristics of the beverage through their heterofermentative metabolism, producing organic acids and ethanol. These species have also shown potential health benefits in other studies (Gomes et al., 2021). *L. spicheri* has been shown to improve food texture and may have a positive effect on intestinal health (Pérez-Alvarado et al., 2022). *L. zymae* is known for its production of *γ*-aminobutyric acid (GABA), a bioactive compound associated with several health benefits (Icer et al., 2024). *Limosilactobacillus* species have been shown to have probiotic properties, including antimicrobial activity, modulation of the immune system and improvement of intestinal barrier function. The production of acetate could be attributed, at least in part, to the activity of the *Acetobacteraceae* family, particularly in relation to this beverage *Acetobacter pasteurianus* which could be isolated as an axenic culture. Noteworthy, 1.6% of the reads were classified as belonging to the genus *Clostridium*, which includes species with the potential to produce harmful toxins (Du et al., 2020; Tyszak and Rehmann, 2024). Certain *Clostridium* species, such as *Clostridium difficile* and *Clostridium perfringens*, are known to produce toxins that can pose health risks, particularly to vulnerable populations such as children and the elderly. However, their relative abundance was generally low, suggesting that their presence alone may not pose an immediate health risk unless conditions are favorable for their proliferation or toxin production. Therefore, our findings highlight the importance of implementing stringent hygiene measures during the manufacturing process to avoid contamination with harmful microorganisms, mainly those of faecal origin (Frew and Abebe, 2020). Some minority groups, including *Lentilactobacillus* (< 1%), were also identified, together with other bacteria potentially derived from the raw materials, such as *Paenibacillus* and *Streptomyces*. Ashenafi and Mehari (1995) and Abegaz et al. (2002) previously observed that during the fermentation of *Borde*, the pH decreased as the number of LABs increased, while the number of *Enterobacteriaceae* and aerobic mesophilic bacteria, such as *Pseudomonas*, decreased. These observations were therefore consistent with the *Borde* sample analyzed in the present study.

### 4.2 Cheka

*Cheka* is an alcoholic fermented drink from south-western Ethiopia, specifically the Dirashe and Konso regions (Wedajo Lemi, 2020). Cereals such as sorghum and maize and vegetables such as kale, moringa and decne are typically included in the composition of this drink. Some households also include dried edible remains of injera, kitta or kurkufa.

The pH content of the Cheka sample analyzed in this study is consistent with previously reported values, ranging from 3.53 to 3.99 for pH, but not for ethanol content ranging from 3.05 to 8.96% (Binitu Worku et al., 2018; Hotessa and Robe, 2020). The wide variation in ethanol content observed in different studies, including this one, may be due to differences in fermentation practices, microbial composition, environmental conditions, and variability in ingredients, all of which can significantly affect the fermentation process and final product characteristics.

In line with this result, the *Cheka* analyzed in our study showed low bacterial diversity, with *Limosilactobacillus*, mainly *Limosilactobacillus panis*, and *Lactobacillus*, mainly *Lactobacillus delbrueckii*, as the dominant bacteria. Many strains of *Lactobacillus delbrueckii* have been shown in previous studies to have a variety of probiotic functions, including immunomodulation. Many strains of *Lactobacillus delbrueckii* have been shown in previous studies to have a variety of probiotic functions, including immunomodulation (Angelescu et al., 2019; Al-Yami et al., 2022; Mo et al., 2022; Wang et al., 2024). However, it is important to note that probiotic properties are strain specific. Therefore, future research is needed to determine the specific probiotic benefits of the *L. delbrueckii* strain found in Cheka fermentation. Unlike the results reported by Gizachew et al. (2023), *Limosilactobacillus fermentum* and *Pediococcus pentosaceus* could not be isolated from our Cheka sample, a discrepancy that could be attributed to the geographical origin of the raw materials and the methods used to prepare them.

### 4.3 Kinito

*Kinito* is a fermented non-alcoholic or low-alcoholic beverage consumed in various regions of Ethiopia, including the Jimma Zone (Tafere, 2015). It is produced by fermenting cereals, mainly barley (Bacha et al., 1999; Hotessa and Robe, 2020). *Kinito* is commonly served during social occasions and provides income for many households.

The *Kinito* sample analyzed showed low levels of ethanol and organic acids with a pH of 3.36, maintaining moderate levels of free sugars possibly due to the short fermentation period. While our analysis showed low levels of the specifically measured organic acids, the low pH of *Kinito* may be due to other organic acids not quantified in this study, such as succinic, malic or tartaric acids. In addition, the initial pH of the barley used in *Kinito* production (typically 5.8–6.2) provides a starting point for mild acidity. The bacterial richness was found to be low, with only two dominant LAB species, *Liquorilactobacillus nagelii* and *Leuconostoc falkenbergense*. The presence of *Enterobacteriaceae* (0.12%) and *Enterococcaceae* (0.02%) in the sample indicates a possible faecal contamination of the raw material, suggesting the need to improve hygiene practices during preparation. A strain of *Leuconostoc pseudomesenteroides* could be isolated *in vitro*, along with representatives of *Liquorilactobacillus*, *Secundilactobacillus*, *Acetobacter*, and *Staphylococcus*. *Leuconostoc* species are recognized for their probiotic potential and ability to produce beneficial compounds (Shin and Han, 2015; Ayalew, 2015).

### 4.4 Shamita

*Shamita* is a fermented drink widely consumed in Ethiopia, particularly by the Gurage community. The fermentation process involves soaking roasted barley flour (Ashenafi and Mehari, 1995). Due to its considerable thickness and high protein content, *Shamita* is often used as a meal replacement supplement (Ashenafi and Mehari, 1995).

The pH of the *Shamita* sample analyzed was 3.4, which was slightly lower than the reported 4.1 (Ashenafi and Mehari, 1995). This discrepancy could be due to a longer time lag between product manufacture and chemical characterization. *Shamita* exhibited the lowest concentrations of ethanol and sugar among the beverages analyzed in this study, with low organic acid levels, mainly lactic acid.

The analyzed sample exhibited low bacterial richness but high diversity in taxa. The most prevalent taxa identified included *Lacticaseibacillus sharpeae*, *Lacticaseibacillus songhuajiangensis*, *Limosilactobacillus pontis*, *Paucilactobacillus vaccinostercus*, *Leuconostoc falkenbergense*, *Levilactobacillus zymae*, and various *Lactobacillus* species. Furthermore, we could isolate seven strains, mainly lactic acid bacteria, in addition to *Peribacillus*, *Staphylococcus*, and *Moraxella*. Strain D12-1 exhibited a 16S rRNA gene similarity of less than 90% to its closest type strain, namely *Lactiplantibacillus plajomi* NB53^T^ (Supplementary Table S2), thereby indicating the potential for the delineation of a novel genus within the family *Lactobacillaceae*. Although Ashenafi and Mehari (1995) reported the presence of aerobic mesophilic bacteria, mainly *Bacillus* spp. and LABs, along with *Enterobacteriaceae* and yeast in other Shamita samples, no aerobic mesophilic bacteria or *Enterobacteriaceae* were identified in the present study.

### 4.5 Tej

*Tej* is a honey wine, both homemade and commercially produced. It contains various ingredients, including honey, plant barks, roots and/or herbal components such as *Olea africana* and Gesho (*Rhamnus prinoides*) (Urga et al., 1997; Wedajo Lemi, 2020).

Bahiru et al. (2001) reported that the physicochemical profile of *Tej* samples can vary between regions as it is highly dependent on the microbial load of the raw material. In particular, the pH of Tej samples can vary between 2.88 and 4.90, although most Tej samples have a pH below 4.0 (Fentie et al., 2022a; Berhanu et al., 2023), the alcohol content ranges from 6.0 to 11.3% (Bahiru et al., 2006; Fentie et al., 2022a; Getachew Fentie, 2022), while the total sugar content ranges from 0.37 to 31.6 g/L according to Fentie et al. (2022a). The pH of the *Tej* analyzed in our study was around 4.0, being the beverage analyzed in this study with the highest ethanol content, namely 45 mg/g and the highest concentration of free sugars.

*Tej* was the second fermented beverage with the highest species richness, although with extremely low Shannon and Simpson diversity index values, indicating the clear numerical dominance of one of the taxa, namely *Zymomonas mobilis*, a member of the *Sphingomonadaceae* family. Other minority members of the community were members of the family *Enterobacteriaceae*, such as *Klebsiella*, and different members of the family *Acetobacteraceae*. Fentie et al. (2022b) carried out a metataxonomic study of the bacterial community of different Tej samples and reported *Lactobacillus* spp. and *Zymomonas mobilis* as the most dominant taxa, with their relative abundance varying between samples depending on their origin. *Zymomonas mobilis* is known for its ability to produce bioethanol and levan-type prebiotics using glucose, fructose, and sucrose as the main carbon sources (Hu et al., 2021). Other minority taxa identified in our Tej sample included members of the genus *Klebsiella*, which constituted 3% of the relative abundance. *Klebsiella* is a genus of gram-negative, facultatively anaerobic bacteria that are part of the normal microbiota of the human gastrointestinal tract, indicating a potential faecal contamination of the raw materials. Certain species, particularly *K. pneumoniae* and *K. oxytoca*, can act as opportunistic pathogens, causing a range of infections, especially in immunocompromised individuals (Podschun and Ullmann, 1998). The gluconic and acetic acids reported in our sample *Tej* could be released by acetic acid bacteria. We were able to isolate four acetic acid-producing strains identified as *Acetobacter tropicalis*, *A. okinawensis*, *A. indonesiensis*, and *A. fabarum*. Other bacteria isolated from *Tej* were a strain of *Moraxella osloensis*, two strains of *Staphylococcus*, and two LABs, *Lacticaseibacillus paracasei* and *Lentilactobacillus hilgardii*. Fentie et al. (2022b) also examined the cultivable fraction of microorganisms from *Tej* samples, with LABs and the yeast *Saccharomyces* being the most isolated microorganisms. Even though our study focused on the analysis of the bacterial community of fermented products of Ethiopian origin, a representative of the genus *Saccharomyces* was also isolated from *Tej* using a culture medium optimized for bacteria, confirming the results of Fentie et al. (2022b) for the presence of yeasts in Tej (data not shown).

### 4.6 Tella

*Tella*, a regionally variable beverage brewed from a variety of grains, typically includes barley and teff, with wheat, sorghum, maize and spices added depending on the region (Lee et al., 2015). The production process of *Tella* mirrors that of beer, incorporating malt and gesho, the latter of which performs a similar function to hops in beer brewing (Getaye et al., 2018; Tekle et al., 2019).

The *Tella* sample in this study had a slightly lower alcohol content than is reported elsewhere, namely 2–6% (Lee et al., 2015; Fentie et al., 2020). The pH of the sample was slightly more acidic, between 4 and 5, compared to other studies (Lee et al., 2015; Fentie et al., 2020). The variation in pH and alcohol content between samples can be influenced by whether or not the beverage has been subjected to filtration (Lee et al., 2015; Fentie et al., 2020). The main organic acids in *Tella* were lactic and acetic acids, with smaller amounts of glutamic and citric acids.

The *Tella* sample had low bacterial richness and diversity. The most abundant genus was *Lactobacillus*, followed by other lactic acid bacteria. LABs were also the main bacterial players in other *Tella* samples (Berza and Wolde, 2014; Tekle et al., 2019; Hotessa and Robe, 2020; Birhanu et al., 2021; Yehuala et al., 2024). LABs also dominated the cultivated fractions of the community, in particular *Lactiplantibacillus*, *Paucilactobacillus*, *Peribacillus*, *Levilactobacillus*, *Lactobacillus*, *Companilactobacillus* and *Lacticaseibacillus*. Other species isolated were *Acetobacter* and *Paenibacillus*. Consistent with our results, Berza and Wolde (2014) and Tekle et al. (2019) isolated the same taxa from other *Tella* samples, including LABs and acetic acid bacteria.

### 4.7 Datta

*Datta*, also known as *Qotchqotcha*, is a spicy chilli paste typically consumed in southern Ethiopia. It is made from green chillies, fresh sweet basil, garlic, ginger and/or rue seeds (Mulaw and Tesfaye, 2017).

The pH of the sample analyzed was within the range of values reported by other authors, typically 4.6–5.6 (Idris and Ashenafi, 2001; Gemechu, 2021). This fermented food had very low concentrations of ethanol, fermentable sugars and organic acids, with citric acid being the main organic acid.

The bacterial community identified in this fermented food condiment was characterized by a relatively high number of different species and a highly homogeneous distribution of their relative abundances. The community was dominated by the family *Marinilabiliaceae* (phylum *Bacteroidota*), whose members have not yet been cultivated (Rosenberg, 2014). The second most abundant genus was *Weissella*, a member of the *Leuconostocaceae* family, which can carry out a heterofermentative metabolism (Fusco et al., 2015). *Weissella* has potential probiotic properties that including antimicrobial and anti-inflammatory effects (Lee et al., 2012; Dey et al., 2019; Fusco et al., 2023), and can produce unique, indigestible sugars that have attracted interest for their prebiotic and industrial uses. Two *Weissella* strains were isolated from the *Datta* sample, along with one strain of *Secundilactobacillus* and *Kocuria*. The next most common genera in the bacterial community were *Rosenbergiella*, *Pseudomonas* (*P. rhizoryzae*), *Bacillus*, *Pantoea* and *Staphylococcus*. Interestingly, no study published to date has shown the dominance of *Weissella* in *Datta*. As Datta is produced by a spontaneous fermentation process, the dominant bacteria after fermentation may be influenced by the origin and nature of the raw materials used. Indeed, previous studies identified *Lactobacillus* spp. and yeasts, mainly *Saccharomyces*, as the dominant taxa in *Datta* (Idris and Ashenafi, 2001; Dandessa, 2019; Wedajo Lemi, 2020).

### 4.8 Injera

*Injera* is a flatbread made from wheat, barley, sorghum, maize and teff, a small round grain that grows in the highlands of Ethiopia. Often referred to as the ‘Ethiopian pancake’, it is served with almost all traditional Ethiopian dishes (Baye et al., 2013; Neela and Fanta, 2020).

The *Injera* sample analyzed in this study had a pH of 3.9, like other samples, namely 3.5–4.0 (Tadesse et al., 2019). Typically, the pH of dough drops from 5.78 to 3.64 after 96 h of fermentation (Tadesse et al., 2019). The sample showed residual amounts of ethanol and free sugars. The amount of organic acids was also not very high, with the fermented product analyzed in this study having the lowest amount of these metabolites. The organic acids reported were mainly acetic acid, followed by lactic acid.

The *Injera* sample was characterized by very low species richness and diversity. In particular, it was dominated by members of the genera *Lactobacillus* and *Limosilactobacillus*, mainly *L. pontis* and *L. panis*. Other minority groups were members of *Levilactobacillus* and *Companilactobacillus*. Metataxonomics studies carried out by Manara et al. (2023) and Ashagrie et al. (2023) identified lactic acid bacteria as the numerically dominant microorganisms, similar to our results. Given that injera is baked at high temperatures (typically above 100°C), most microorganisms present in the dough would be eliminated during cooking. However, the presence of microbial taxa detected through metataxonomic analysis can be attributed to post-baking contamination from handling surfaces, utensils, air exposure, or packaging materials. Additionally, as metataxonomics detects DNA fragments rather than only viable microorganisms, some non-viable bacterial DNA from fermentation-associated microbes, such as lactic acid bacteria, may persist even after baking and still be amplified during sequencing.

Four different strains of the genus *Acetobacter* could be isolated from our *Injera* sample, although members of this genus were a minority in the sample analyzed (data not shown). These acetogenic bacteria, together with LAB, would be responsible for the organic acid profile detected in the *Injera* sample. Previous culture-dependent studies identified *Pediococcus pentosaceus*, *Limosilactobacillus fermentum* and *Lactiplantibacillus plantarum* as members of the bacterial community in *Injera* (Desiye and Abegaz, 2013; Fischer et al., 2014; Tadesse et al., 2019). Notably, no members of the genera *Pediococcus* or *Leuconostoc* were detected in our study, either by mass sequencing or culturomics. Yeasts such as *Saccharomyces cerevisiae*, *Candida humilis* and *Pichia fermentans* also play an essential role in the fermentation process (Tadesse et al., 2019; Muche et al., 2023). These yeasts could be responsible for the production of carbon dioxide, which forms the characteristic holes in injera and contributes to its spongy texture.

### 4.9 Kotcho

*Kotcho* is a fermented food made from the pseudostem and corm of the enset plant (*Ensete ventricosum*) (Yirmaga, 2013; Dibaba et al., 2018; Birmeta et al., 2019). The quality of *Kotcho* depends on the enset variety, fermentation time and processing methods (Yirmaga, 2013; Karssa et al., 2014).

The pH decreases significantly during fermentation, reaching 3.5–4.0 (Dekeba Tafa and Abera Asfaw, 2020), which is consistent with our observation. *Kotcho* had high amount of organic acids, mainly acetic and lactic acids, with traces of L-glutamic acid.

At the microbiological level, the *Kotcho* sample analyzed showed a high taxonomic diversity, both in terms of richness and Shannon and Simpson indices, indicating the presence of a large number of species with an even distribution of species abundance. The species *Secundilactobacillus paracollinoides*, *Schleiferilactobacillus harbinensis* and *Lactiplantibacillus* spp. were the numerically dominant bacteria. *Lactiplantibacillus plantarum* was the most frequently isolated LAB species by Birmeta et al. (2019) in different *Kotcho* samples. In line of this result, we could isolate a strain of *Lactiplantibacillus plantarum*. *Bifidobacterium minimum* and *Bifidobacterium mongoliense* accounted for 5.0 and 1.5% of the total community, respectively. Although *Leuconostoc mesenteroides* is considered to be the bacterial initiator of *Kotcho* fermentation (Dibaba et al., 2018), *Leuconostoc* made up less than 1% in our *Kotcho* sample, possibly because the sample analyzed was at an advanced stage of fermentation and could have been outcompeted by other lactic acid bacteria. Despite the amounts of acetic acid in the *Kotcho* sample, members of the genus *Acetobacter* accounted for less than 1%. In our culturomic analysis we were able to isolate a strain of *Acetobacter*. Weldemichael et al. (2019) proposed that the concentration of *Acetobacter* is dependent on the starter culture added at the beginning of the fermentation period. We did not observe any members of *Enterobacteriaceae*, in line with the findings of Karssa et al. (2014) and Dibaba et al. (2018), due to the unfavorable conditions resulting from the decrease in pH during fermentation.

## 5 Final remarks

This study provides a comprehensive investigation of the microbial ecology of representative traditional Ethiopian fermented foods, condiments and beverages, highlighting the diversity of microorganisms involved in fermentation processes and how they may represent a potential source of probiotics. By combining culturomic and metataxonomic techniques with physicochemical characterization of the samples, we identified both the predominant and key lactic acid bacteria, as well as the less common microbial species involved in the fermentation. To date, most microbiological studies have relied on culture-dependent techniques, limiting knowledge to those bacterial taxa that can be cultured *in vitro* and/or are numerically dominant in communities. Our study sheds light on the microbial ecology of fermented foods, condiments and beverages from a holistic perspective.

It is important to emphasize that in this study we only analyzed a single sample of each fermented product, with the aim of providing an initial insight into the microbial communities present in a range of traditional Ethiopian fermented foods and beverages. While this approach allowed us to identify a diversity of microorganisms associated with these products, it does not capture the potential variability due to differences in geographical origin or preparation methods. Therefore, future research should include multiple samples of each product, collected from different regions and producers, to gain a more comprehensive understanding of the biogeography and dynamics of the microbial communities involved.

The relative abundance of bacterial species varied significantly between the different fermented products analyzed. Lactic acid bacteria were dominant in most products, particularly in beverages such as *Cheka* and *Tella* and in foods such as Injera. In contrast, other taxonomic groups predominated in certain products, e.g., *Zymomonas mobilis* dominated *Tej* and *Weissella* species dominated *Datta*. Our findings highlight the different microbial communities that drive the fermentation processes in each product, which may influence their fermentation processes and potential health effects. We found that the bacterial species involved in the fermentation processes could contribute to the safety and nutritional quality of fermented foods, and based on previous studies, may also play a key role in enhancing their potential probiotic properties (Quinto et al., 2014; Zielińska and Kolożyn-Krajewska, 2018). However, we did not have a direct assessment of the probiotic properties of the isolates in our study and this will be the subject of future research. The rich diversity of LABs, particularly in products such as *Borde*, *Shamita* and *Injera*, suggests significant health benefits, including their probiotic potential. The presence of unique microbial communities in *Tej*, *Kotcho* and *Datta* demonstrates the variability of fermentation processes depending on substrates and regional practices. Future research should focus on deeper functional analyses of the microorganisms involved, particularly their probiotic potential and health implications. In addition, exploring the scalability and standardization of traditional fermentation practices for wider food production could help address food security challenges in developing regions. The presence of potential bacterial pathogens in *Cheka*, *Borde* and *Tej* highlights the dual nature of fermented foods and beverages: while they can serve as a source of probiotics, they can also pose microbiological risks if not properly managed (Nemo and Bacha, 2021). Ensuring the safety of fermented products requires a thorough understanding of the microbial dynamics involved and the implementation of good manufacturing practices. To mitigate microbiological risks, traditional fermented products can benefit from improved hygiene practices, strict control of fermentation parameters and pre-fermentation treatments such as pasteurization.

## Data Availability

The datasets from this study are available in a public data repository: PRJNA1218641.
