# Supplementary material for: Microbial ecology of selected traditional Ethiopian fermented products
Source: Front Microbiol. 2025 Jun 2;16:1570914. doi: 10.3389/fmicb.2025.1570914 (PMC12171217; doi:10.3389/fmicb.2025.1570914)
Supplement: Supplementary file 1 [file Data_Sheet_1.pdf]

# Supplementary Material

# Microbial Ecology of Selected Traditional Ethiopian Fermented Products

Carmen Sanz-López[a#], Michela Amato[a#], Daniel Torrent[a], Marta Borrego[a], Mathewos Anza[c], Mesfin Bibiso[c], Nubia Grijalva-Vallejos[d], Cristina Vilanova[a], Manuel Porcar[a,b] and Javier Pascual[a]

[a]Darwin Bioprospecting Excellence S.L., Paterna, Spain

[b]Institute for Integrative Systems Biology I2SysBio (University of Valencia - CSIC), Paterna, Spain.

[c]Department of Chemistry, College of Natural and Computational Sciences, Wolaita Sodo University, Wolaita Sodo, Ethiopia

[d]Quiitos S.A.S, San Antonio de Ibarra, Ecuador

#These authors have contributed equally to this work

*Corresponding authors: Javier Pascual, jpascual@darwinbioprospecting.com

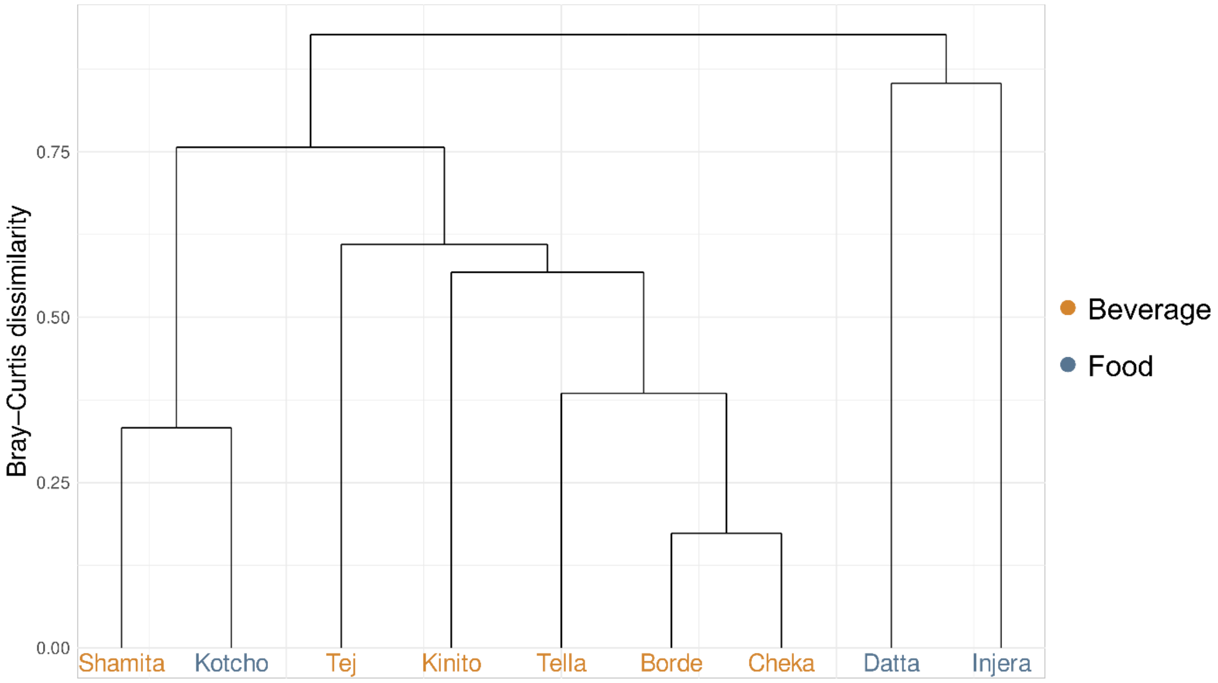


**Supplementary Figure S1.** UPGMA dendrogram of nutrient profiles. This figure shows a UPGMA dendrogram based on the Bray-Curtis similarity of the nutrient profiles, including free sugars, alcohol and organic acids, in the fermented foods, including a condiment, and beverages studied**.**

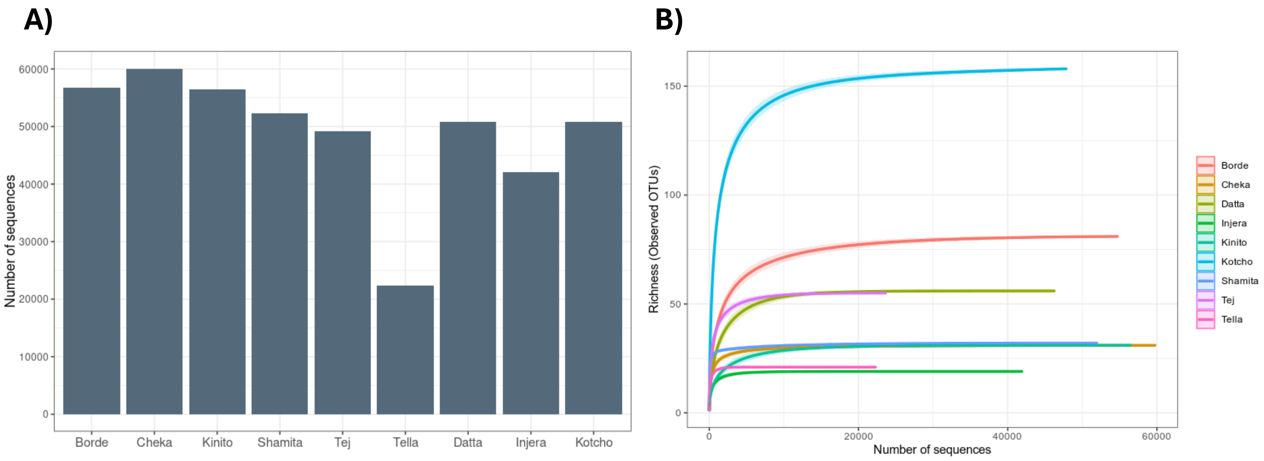


**Supplementary Figure S2.** (A) Number of high-quality reads obtained for each fermented sample. (B) Rarefaction curves of the samples.

**Supplementary Table 1.** Relative Abundances (%) of ASV in Nine Fermented Products. This table presents the relative abundances (%) of Amplicon Sequence Variants (ASV) in the nine fermented products. The last taxonomic level at which representatives were classified is shown (Sheet 2 Excel file).

**Supplementary Table 2.** Taxonomic Affiliation of the Strains Isolated from Traditional Foods and Beverages Based on their 16S rRNA gene Sequence Similarity.

| Strain | Fermented product | Closest type strain (Accession number) | Similarity (%) |
|---|---|---|---|
| D9-2 | *Tej* | *Moraxella osloensis* A1920$^{T}$ (NR_104936.1) | 99.22 |
| D9-3 | *Tej* | *Staphylococcus hominis subsp. novobiosepticus* GTC 1228$^{T}$ (NR_041323) | 99.80 |
| D9-5 | *Tej* | *Staphylococcus epidermidis* strain *Fussel*$^{T}$ = NBRC 100911$^{T}$ | 99.68 |
| D9-7 | *Tej* | *Acetobacter okinawensis* JCM 25146 1-35$^{T}$ (NR_113546.1) | 99.90 |
| D9-8 | *Tej* | *Acetobacter indonesiensis* NBRC 16471$^{T}$ *(*NR_113847.1*)* | 99.90 |
| D9-10 | *Tej* | *Acetobacter fabarum* R-36330$^{T}$ (NR_113556.1) | 100 |
| D9-14 | *Tej* | *Lacticaseibacillus paracasei* ATCC 25302$^{T}$ (NR_113337.1) | 99.67 |
| D9-16 | *Tej* | *Acetobacter tropicalis* NBRC 16470$^{T}$ (NR_113846.1) | 100 |
| D9-19 | *Tej* | *Lentilactobacillus hilgardii* NBRC 15886$^{T}$ (NR_113817.1) | 99.86 |
| D9-20 | *Tej* | *Saccharomyces cerevisiae* | 99.85 |
| | | | |
| D10-1 | *Borde* | *Paenibacillus polymyxa* DSM 36$^{T}$ (NR_114810.1) | 99.59 |
| D10-5 | *Borde* | *Acetobacter pasteurianus subsp. pasteurianus* LMG 1262 = NBRC 106471$^{T}$ (NR_118169.1) | 99.77 |
| D10-6 | *Borde* | *Streptomyces coelicolor* DSM 40233$^{T}$ (NR_116633.1) | 99.67 |
| D10-32 | *Borde* | *Kazachstania humilis* | 99.66 |
| D10-9 | *Borde* | *Paenibacillus ottowii* MS2379$^{T}$ (NR_180200.1) | 99.11 |
| D10-14 | *Borde* | *Moraxella osloensis* A1920$^{T}$ (NR_104936.1) | 99.10 |
| D10-15 | *Borde* | *Lacticaseibacillus paracasei* NBRC 15889$^{T}$ (NR_113337.1) | 99.89 |
| D10-23 | *Borde* | *Lentilactobacillus parabuchneri* JCM 12493$^{T}$ (NR_041294.1) | 99.79 |
| D10-27 | *Borde* | *Lentilactobacillus hilgardii* NBRC 15886$^{T}$ (NR_113817.1) | 100 |
| D10-31 | *Borde* | *Companilactobacillus paralimentarius* DSM 13238$^{T}$ (NR_114844.1) | 99.88 |
| D10-33 | *Borde* | *Lentilactobacillus diolivorans* JKD6$^{T}$ (NR_037004.1) | 99.90 |
| D10-46 | *Borde* | *Lentilactobacillus buchneri* JCM 1115$^{T}$ (NR_041293.1) | 99.79 |
| | | | |
| D11-1 | *Kinito* | *Acetobacter fabarum* LMG 24244$^{T}$ (NR_113556.1) | 100 |
| D11-5 | *Kinito* | *Leuconostoc pseudomesenteroides* KCTC 3652 LMG 11482T (NR_109004.1) | 99.90 |
| D11-13 | *Kinito* | *Secundilactobacillus collinoides* JCM1123$^{T}$ (NR_024645.1) | 99.57 |
| D11-23 | *Kinito* | *Staphylococcus condimenti* CIP105760$^{T}$ (NR_116435.1) | 100 |
| D11-24 | *Kinito* | *Liquorilactobacillus nagelii* LuE10$^{T}$ (NR_119275.1) | 99.90 |

| Strain | Fermented product | Closest type strain (Accession number) | Similarity (%) |
|---|---|---|---|
| | | | |
| D12-1 | *Shamita* | *Lactiplantibacillus plajomi* NB53$^T$ (NR_136785.1) | 89.34 |
| D12-2 | *Shamita* | *Moraxella osloensis* DSM 6998$^T$ (NR_113392.1) | 99.58 |
| D12-8 | *Shamita* | *Solibacillus silvestris* HR3-23$^T$ (NR_028865.1) | 100 |
| D12-14 | *Shamita* | *Staphylococcus pseudoxylosus* S04009$^T$ (NR_180150.1)/ *Staphylococcus saprophyticus subsp. saprophyticus* ATCC 15305$^T$ = NCTC 7292$^T$ (NR_074999.2) | 100 |
| D12-26 | *Shamita* | *Lactiplantibacillus plantarum* JCM 1149$^T$ (NR_115605.1) | 100 |
| D12-31 | *Shamita* | *Levilactobacillus zymae* R-18615$^T$ (NR_042241.1) | 99.69 |
| D12-7 | *Shamita* | *Lactiplantibacillus fabifermentans* DSM 21115$^T$ (NR_113339.1) | 99.53 |
| | | | |
| D13-2 | *Kotcho* | *Paenibacillus seodonensis* DCT-19$^T$ (NR_165702.1) | 99.57 |
| D13-3 | *Kotcho* | *Lysinibacillus mangiferihumi* M-GX18$^T$ (NR_118146.1) | 98.89 |
| D13-9 | *Kotcho* | *Acetobacter indonesiensis* NBRC 16471$^T$ (NR_113847.1) | 99.90 |
| D13-11 | *Kotcho* | *Paenibacillus dongdonensis* KUDC0114$^T$ (NR_134112.1) | 99.57 |
| D13-14 | *Kotcho* | *Microbacterium ginsengisoli* Gsoil 259$^T$ (NR_041516.1) | 97.09 |
| D13-29 | *Kotcho* | *Secundilactobacillus paracollinoides* DSM 15502$^T$ (NR_042322.1) | 99.70 |
| D13-40 | *Kotcho* | *Lentilactobacillus kisonensis* YIT 11168$^T$ (NR_041658.1) | 99.30 |
| D13-46 | *Kotcho* | *Lactiplantibacillus plantarum* JCM 1149$^T$ (NR_115605.1) | 99.90 |
| D13-59 | *Kotcho* | *Lactiplantibacillus fabifermentans* DSM 21115$^T$ (NR_113339.1) | 100 |
| D13-27 | *Kotcho* | *Pichia occidentalis* | 100 |
| D13-41 | *Kotcho* | *Staphylococcus haemolyticus* JCM 2416$^T$ | 99.39 |
| D13-44 | *Kotcho* | *Staphylococcus hominis subsp. novobiosepticus* GTC 1228$^T$ | 99.14 |
| D13-6 | *Kotcho* | *Streptomyces coelicolor* DSM 40233$^T$ (NR_119342.1) | 99.15 |
| D13-15 | *Kotcho* | *Leuconostoc miyukkimchii* M2$^T$ (NR_109072.1) | 99.18 |
| | | | |
| D14-1 | *Tella* | *Paenibacillus humicus* PC-147$^T$ (NR_042577.1) | 99.48 |
| D14-2 | *Tella* | *Paenibacillus seodonensis* DCT-19$^T$ (NR_165702.1) | 99.54 |
| D14-5 | *Tella* | *Lactiplantibacillus fabifermentans* DSM 21115$^T$ (NR_113339.1) | 100 |
| D14-6 | *Tella* | *Paucilactobacillus vaccinostercus* NRIC 1075$^T$ (NR_112541.1) | 100 |
| D14-8 | *Tella* | *Acetobacter indonesiensis* NBRC 16471$^T$ (NR_113847.1) | 99.9 |
| D14-15 | *Tella* | *Peribacillus acanthi* L28$^T$ (NR_179899.1) | 99.88 |
| D14-20 | *Tella* | *Acetobacter fabarum* LMG 24244$^T$ (NR_113556.1) | 99.81 |

| Strain | Fermented product | Closest type strain (Accession number) | Similarity (%) |
|---|---|---|---|
| D14-21 | *Tella* | *Levilactobacillus brevis* ATCC 14869$^{T}$ = DSM 20054$^{T}$ (NR_116238.1) | 100 |
| D14-23 | *Tella* | *Companilactobacillus paralimentarius* DSM 13238$^{T}$ (NR_114844.1) | 99.72 |
| D14-24 | *Tella* | *Lactiplantibacillus pentosus* 124-2$^{T}$ (NR_029133.1)/ *Lactiplantibacillus plantarum* CIP 103151$^{T}$ (NR_104573.1) | 99.90 |
| D14-26 | *Tella* | *Lacticaseibacillus paracasei* ATCC 25302$^{T}$ (NR_117987.1) | 100 |
| D14-32 | *Tella* | *Lactobacillus helveticus* NBRC 15019$^{T}$ (NR_113719.1) | 99.54 |
| D14-33 | *Tella* | *Lacticaseibacillus pantheris* LMG 21017$^{T}$ (NR_025189.1) | 99.88 |
| | | | |
| D15-4 | *Cheka* | *Acetobacter indonesiensis* NBRC 16471$^{T}$ (NR_113847.1) | 99.90 |
| D15-7 | *Cheka* | *Lacticaseibacillus paracasei* R094$^{T}$ (NR_025880.1) | 100 |
| D15-18 | *Cheka* | *Lacticaseibacillus paracasei* R094$^{T}$ (NR_025880.1) | 99.90 |
| D15-9 | *Cheka* | *Lentilactobacillus buchneri* JCM 1115$^{T}$ (NR_041293.1) | 99.89 |
| D15-11 | *Cheka* | *Lentilactobacillus diolivorans* JKD6$^{T}$ (NR_037004.1) | 99.77 |
| D15-30 | *Cheka* | *Lentilactobacillus kisonensis* YIT 11168$^{T}$ (NR_041658.1) | 99.68 |
| | | | |
| D16-2 | *Datta* | *Weissella sagaensis* X0750$^{T}$ (NR_175448.1) | 100 |
| D16-3 | *Datta* | *Weissella cibaria* II-I-59$^{T}$ (NR_036924.1) | 100 |
| D16-8 | *Datta* | *Kocuria palustris* TAGA27$^{T}$ (NR_026451.1) | 100 |
| D16-11 | *Datta* | *Secundilactobacillus collinoides* JCM1123$^{T}$ (NR_024645.1) | 99.76 |
| | | | |
| D17-1 | *Injera* | *Acetobacter fabarum* LMG 24244$^{T}$ (NR_113556.1) | 100 |
| D17-6 | *Injera* | *Acetobacter okinawensis* JCM 25146 1-35$^{T}$ (NR_113546.1) | 99.88 |
| D17-10 | *Injera* | *Acetobacter tropicalis* NBRC 16470$^{T}$ (NR_113846.1) | 100 |
| D17-13 | *Injera* | *Brevibacillus borstelensis* NBRC 15714$^{T}$ (NR_113799.1) | 100 |
| D17-14 | *Injera* | *Peribacillus acanthi* L28$^{T}$ (NR_179899.1) | 100 |
| D17-16a | *Injera* | *Lacticaseibacillus paracasei* R094$^{T}$ (NR_025880.1) | 100 |
| D17-18 | *Injera* | *Lentilactobacillus kisonensis* YIT 11168$^{T}$ (NR_041658.1) | 99.58 |
| D17-25 | *Injera* | *Acetobacter indonesiensis* NBRC 16471$^{T}$ (NR_113847.1) | 100 |
